# Fluorescent Multifunctional Organic Nanoparticles for Drug Delivery and Bioimaging: A Tutorial Review

**DOI:** 10.3390/pharmaceutics14112498

**Published:** 2022-11-17

**Authors:** Guillem Vargas-Nadal, Mariana Köber, Audrey Nsamela, Francesca Terenziani, Cristina Sissa, Silvia Pescina, Fabio Sonvico, Amirah Mohd Gazzali, Habibah A. Wahab, Luca Grisanti, María Eugenia Olivera, María Celeste Palena, María Laura Guzman, Laura Carolina Luciani-Giacobbe, Alvaro Jimenez-Kairuz, Nora Ventosa, Imma Ratera, Kevin D. Belfield, Ben M. Maoz

**Affiliations:** 1 Department of Chemistry, Life Sciences and Environmental Sustainability, University of Parma, Parco Area delle Scienze 17A, 43124 Parma, Italy; 2 Institut de Ciència de Materials de Barcelona (ICMAB-CSIC), 08193 Bellaterra, Spain; 3 Centro de Investigación Biomédica en Red de Bioingeniería, Biomateriales y Nanomedicina, Instituto de Salud Carlos III, 08193 Bellaterra, Spain; 4 Elvesys SAS, 172 Rue de Charonne, 75011 Paris, France; 5 Chair of Physical Chemistry, TU Dresden, 01062 Dresden, Germany; 6 ADDRes Lab, Department of Food and Drug, University of Parma, Parco Area delle Scienze 27A, 43124 Parma, Italy; 7 School of Pharmaceutical Sciences, Universiti Sains Malaysia, USM, Gelugor 11800, Penang, Malaysia; 8 Division of Theoretical Physics, Ruđer Bošković Institute, Bijenička Cesta 54, 10000 Zagreb, Croatia; 9 Unidad de Investigación y Desarrollo en Tecnología Farmacéutica (UNITEFA), CONICET and Departamento de Ciencias Farmacéuticas, Facultad de Ciencias Químicas, Universidad Nacional de Córdoba, Ciudad Universitaria, Córdoba 5000, Argentina; 10 Department of Chemistry and Environmental Science, New Jersey Institute of Technology, 323 Martin Luther King Jr. Blvd., Newark, NJ 07102, USA; 11 Department of Biomedical Engineering, Tel Aviv University, Tel Aviv 6997801, Israel; 12 Sagol School of Neuroscience, Tel Aviv University, Tel Aviv 6997801, Israel; 13 The Center for Nanoscience and Nanotechnology, Tel Aviv University, Tel Aviv 6997801, Israel

**Keywords:** fluorescence, organic nanoparticles, drug delivery, bioimaging, fluorophores, fluorescence microscopy, theoretical simulations of nanoparticles, FONs manufacturing

## Abstract

Fluorescent organic nanoparticles (FONs) are a large family of nanostructures constituted by organic components that emit light in different spectral regions upon excitation, due to the presence of organic fluorophores. FONs are of great interest for numerous biological and medical applications, due to their high tunability in terms of composition, morphology, surface functionalization, and optical properties. Multifunctional FONs combine several functionalities in a single nanostructure (emission of light, carriers for drug-delivery, functionalization with targeting ligands, etc.), opening the possibility of using the same nanoparticle for diagnosis and therapy. The preparation, characterization, and application of these multifunctional FONs require a multidisciplinary approach. In this review, we present FONs following a tutorial approach, with the aim of providing a general overview of the different aspects of the design, preparation, and characterization of FONs. The review encompasses the most common FONs developed to date, the description of the most important features of fluorophores that determine the optical properties of FONs, an overview of the preparation methods and of the optical characterization techniques, and the description of the theoretical approaches that are currently adopted for modeling FONs. The last part of the review is devoted to a non-exhaustive selection of some recent biomedical applications of FONs.

## 1. Introduction

**Figure 1:**
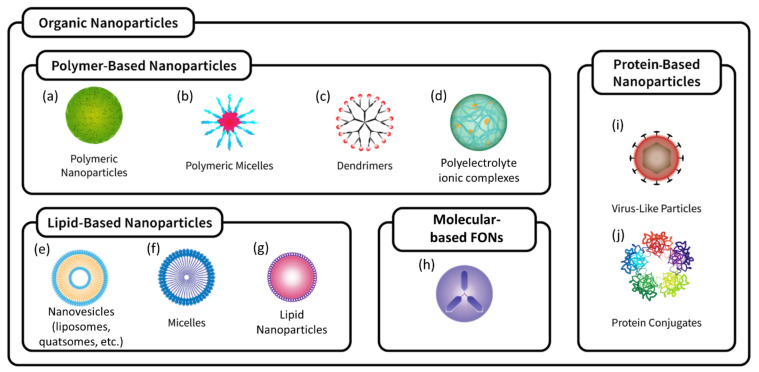
Schematic representation of common organic nanoparticles classified with respect to their composition: polymer-based nanoparticles ((**a**) polymeric nanoparticles, (**b**) polymeric micelles, (**c**) dendrimers, (**d**) polyelectrolyte ionic complexes); lipid-based nanoparticles ((**e**) nanovesicles, (**f**) micelles, (**g**) lipid nanoparticles); molecular-based FONs (**h**); protein-based nanoparticles ((**i**) virus-like particles, (**j**) protein conjugates).

Nanoparticles (NPs) have proven to be a valuable tool in biochemistry, pharmaceutical, and biomedical sciences, as the sub-micrometer size of the particles enables the appearance of unique features, which are significantly different from bulk materials, including physicochemical, biochemical, magnetic, optical, and electronic properties [1,2,3,4,5]. Moreover, these materials have a high surface-area-to-volume ratio and, due to their small size and specific chemical properties, have been used for many applications in biomedical fields, such as drug delivery, imaging, and diagnostics [3,5]. In recent years, the number of reviews addressing the applications of NPs in bioimaging and drug delivery, with a focus on the enhancement of optical properties (such as chirality [6], Raman [7], and others [8,9]), has progressively increased. In this review, we will focus on organic NPs, and a non-exhaustive overview of organic NPs is presented in Figure 1.

**Figure 2:**
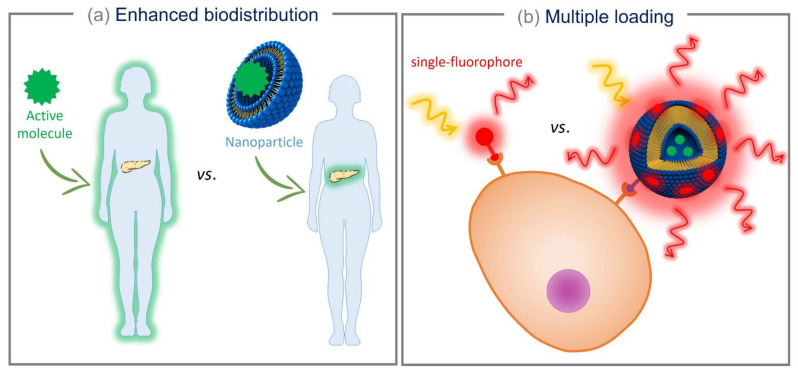
Schematic representation of two of the advantages of using nanoparticles for drug delivery and bioimaging. (**a**) Enhanced biodistribution due to passive or active tissue targeting. (**b**) Multiple fluorophores loading with the related brightness increase.

For drug delivery applications, NPs are specifically optimized for different administration routes and are primarily used to increase the solubility and stability of drugs. Incorporating hydrophobic drugs into appropriately designed NPs can improve their bioavailability upon administration [10,11,12]. Another advantage of using NPs in the nanomedicine field is related to their impact on the pharmacokinetics of encapsulated drugs or active molecules in the body. Upon parenteral administration, small biologically active molecules are usually rapidly eliminated through biliary clearance and through renal clearance, via the glomerular filtration [13]. On the contrary, when encapsulated in surface-modified NPs, small biologically active molecules show an increased circulation time, affording long blood circulation times. Moreover, NPs can be functionalized with specific moieties to facilitate the delivery of the drug to specific targets [14,15]. In particular, the NP can interact with specific cell receptors, promoting the internalization of the drug and directing its action on the targeted cells (Figure 2a).

Furthermore, NPs can protect the drug or the therapeutic agent from enzymatic degradation, from immune system cells, or from certain proteins (e.g., complement system proteins) normally contained in the bloodstream [16,17], thus increasing the effectiveness of the drug. Eventually, NPs can be loaded with multiple drugs and/or fluorescent molecules, enhancing their therapeutic effect, the signal produced for bioimaging, or even obtaining a theranostic effect [18,19,20] (Figure 2b). On the other hand, if the NPs are administered via a local route (topical, nasal, pulmonary, or ocular delivery), an enhanced permeation of the drug through the biological barriers [21,22] and a protection of the drug from innate defensive proteins or enzymes [23] can be achieved.

In this review, we will focus on fluorescent (multifunctional) organic nanoparticles (FONs), a specific type of NPs, mainly composed of natural or synthetic organic molecules/polymers, and on their use for drug delivery and bioimaging. In the first part of the review, we will present a short overview of different types of organic nanoparticles reported in recent years, and their unique properties. Then, we will briefly present the FONs’ manufacturing, characterization, modeling, and applications in drug delivery and bioimaging.

## 2. General Aspects for FON Design

**Figure 3:**
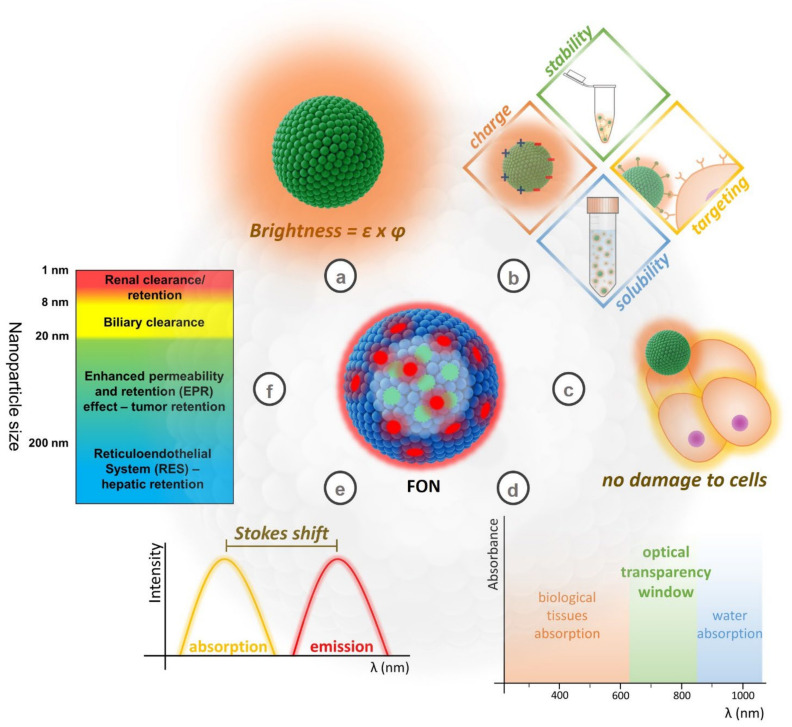
Main features of FONs required for bioimaging application: (**a**) high brightness; (**b**) adequate physicochemical properties; (**c**) low cytotoxicity; (**d**) absorption and emission in the NIR; (**e**) high Stokes shift; (**f**) opportune size.

The ideal fluorescent probe for bioimaging must fulfill several requirements, as shown in Figure 3 [2,24]. Fulfilling all these requirements represents a tough challenge: that is why many classes of fluorescent probes are investigated in the literature for use in fluorescence microscopy.

**Figure 4:**
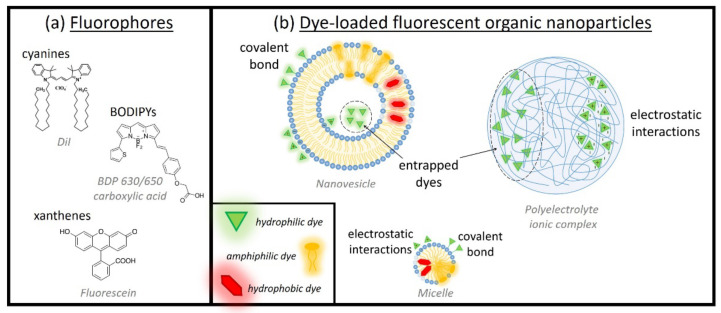
(**a**) Chemical structure of several fluorophores commonly used for FON preparation. (**b**) Schematic representation of different strategies to stably integrate fluorescent molecules into organic nanoparticles.

The colloidal stability of FONs in water and biological fluids is an extremely important aspect, especially in the case of in vivo imaging, e.g., when examining blood vessels and circulation [25,26], or to obtain a suitable accumulation of the probes in tumors. An important conceptual difference exists between dye- and drug-loaded nanoparticles used in imaging and drug release applications, respectively. In the latter case, the encapsulated drug is meant to be released in the surrounding medium (e.g., tumor cells or some specific organelle), while in the case of dye-loaded FONs, the release of the fluorophore is undesired. Fluorophore leaching can decrease the brightness of the nanoparticle, while increasing the background signal [27]. Dye leaching is especially problematic in the formulations in which the dye is physically entrapped in a matrix. A large variety of fluorophores is commercially available as contrast agents for fluorescence bioimaging that belong to different classes of organic dyes (cyanines, BODIPYs, squaraines, xanthenes, etc.). Some examples of molecular structures of different families of fluorophores are reported in Figure 4, together with the different anchorings/interactions with nanoparticles.

In the case of FONs containing several fluorophores, their confinement in the nanosized structure brings about intermolecular interactions that can have different consequences on the fluorescence properties. When the intermolecular interactions are strong, two different scenarios can occur. In most cases, aggregation-caused quenching (ACQ) is observed, so that the FONs are poorly emissive despite being composed of or containing several fluorophores [28]. A simple strategy to prevent ACQ is the use of fluorophores with a bent chemical structure or functionalized with sterically bulky groups [29,30,31,32] or polymer chains [33] that hinder the close π-π stacking of the chromophores. In other (uncommon) cases, an aggregation-induced emission (AIE) is observed, i.e., the emission quantum yield is higher in the aggregate than in the single molecule [34,35]. For this to occur, the molecules constituting the NP have to “light up” when forming aggregates, thanks to restriction of intramolecular motion, which promotes radiative decay [36].

The ability of the fluorophore to form H- or J-aggregates must also be considered: when the intermolecular interactions are particularly strong, excited states become delocalized, strongly affecting the absorption and emission spectra of the fluorophore. H-aggregates are typically non-fluorescent, while J-aggregates can be strongly fluorescent [37].

Other specific requirements characterize each type of NPs. For example, when the reprecipitation method is used to obtain small-molecule FONs, the molecular fluorophore must be highly soluble in an organic solvent miscible with water (water will act as non-solvent) [38]. In dye-loaded NPs, the fluorophore must be chosen not only accounting for its intrinsic fluorescent properties, but also considering its physicochemical affinity with the specific nanocarrier constituents. For example, lipidic nanoparticles are usually loaded with organic lipophilic fluorophores [9], i.e., fluorophores that are chemically modified to conjugate the chromophoric core with long alkyl chains (but preserving emissive properties) [39]. In polyelectrolytes, dye loading can be achieved by exploiting different physicochemical interactions, including ionic interaction, π-π stacking, hydrogen bonds, covalent bonds, or the formation of inclusion complexes [40].

## 3. Overview of Fluorescent Organic Nanoparticles

Various approaches have been employed to produce FONs with different structures. The most common is the loading of fluorescent molecules into non-fluorescent nanoparticle structures (such as nanovesicles, micelles, polymeric nanoparticles, etc., Figure 1), either through covalent bonding or electrostatic/hydrophobic interactions. An alternative approach is the direct use of fluorescent molecules as the main building blocks of the nanoparticle’s structure, for example, by forming nanovesicles from amphiphilic fluorescent molecules, or organic nanoparticles from the self-association of fluorescent molecules. In the following, several examples are provided.

### 3.1. FONs Exclusively Composed by Fluorescent Molecules

#### 3.1.1. Small-Molecule FONs

Nanoparticles purely composed of small organic fluorophores (Figure 1h) can be obtained either in a top–down approach by mechanical milling, ultrasound, or laser ablation of the raw materials [41,42], or in a bottom–up approach by the conversion of the products dissolved in suitable solvents into nano-dispersed systems by reprecipitation in a non-solvent [43,44,45]. They can be obtained as nanocrystals or, more commonly, as amorphous nanostructures. These fluorescent nanoaggregates have been demonstrated to work as brilliant one- and two-photon emitters for bioimaging [46]. They can also be obtained as multicomponent nanosystems, either via the simultaneous reprecipitation of two or more different molecules (each nanoparticle contains a mixture of the different dyes) or via subsequent reprecipitation processes, facilitating the production of totally organic multi-shell nanoparticles with unique fluorescence properties thanks to the interfacial effects and Föster Resonance Energy Transfer (FRET) [47,48].

#### 3.1.2. Nanovesicles Formed by Amphiphilic Fluorescent Molecules

In order to achieve high fluorophore loading, amphiphilic dyes have been developed to facilitate the supramolecular self-assembly into nanovesicles, enabling activated fluorescence and photoacoustic imaging. The Zheng group has demonstrated the self-assembly of porphyrin–lipid conjugates into so-called “porphyrsomes”, spherical vesicles approx. 100 nm in diameter that showed strong fluorescence self-quenching and enabled the sensitive visualization of lymphatic systems using photoacoustic tomography, as well as near-infrared fluorescence generation upon vesicle dissociation [49]. In a similar approach, the same group synthesized an aza-BODIPY-lipid building block that self-assembled into “BODIPYsome” vesicles, exhibiting stable NIR J-aggregation and high fluorescence quenching efficiency. Upon intravenous injection in mice bearing orthotopic prostate cancer, fluorescence was observed at the tumor location 6 and 24 h post-injection, indicating the gradual “BODIPYsome” accumulation and reduction in fluorescence quenching due to nanoparticle disruption [50].

### 3.2. FONs Constituted by Non-Fluorescent Nanoparticles Loaded with Fluorescent Molecules

#### 3.2.1. Polyelectrolyte Ionic Complexes

Polyelectrolytes (PEs, Figure 1d) are hydrophilic polymers containing ionizable groups, which can dissociate, generating charged polymer chains (macroions) and small counterions when dispersed in a polar solvent [51]. PEs can be anionic, with carboxylate, phosphate, and sulfonate groups as ionizable moiety, or cationic, in most cases due to the presence of primary, secondary, and quaternary amino groups. PEs can exhibit both the properties of polymers and electrolytes, which is advantageous toward their interactions with several types of oppositely charged molecules [51]. For these reasons, natural (sodium alginate, chitosan), semi-synthetic (cellulose derivatives), and synthetic (polyacrylic acid) PEs are extensively investigated for different fields of application, including the biomedical field and drug delivery [52]. In fact, the interaction of water-soluble PEs with counterions, such as pharmaceutical drugs or dyes for bioimaging [40,53], generates stable self-assembled colloidal dispersions [54] or discrete nanoparticles [55,56] in which a high proportion of the counterion is electrostatically attached to the PEs, forming polyelectrolyte complexes (PECs) [52]. The PECs system behaves as a carrier, with a high proportion of drug/dye reversibly attached to the PEs as ionic pairs. With organic counterions, non-electrostatic contributions, such as hydrogen bonding and hydrophobic interactions, would also play a role in the association process [55,57,58]. The ion-pair equilibrium in PECs is partially displaced by the addition of salts through ionic exchange but is not modified by the addition of non-electrolytes [59]. As a result, they can provide a sustained release of drug/dye in biological fluids.

The addition of a second, oppositely charged PE (as counter PE) to a binary PEC, can produce drug-interpolyelectrolyte complexes. As with PECs, a remarkably high proportion of the drug/dye is condensed with the PEs in the form of ionic pairs, leading to slow drug release. The release rate can be increased in a saline solution due to the ionic exchange. Contrary to PECs, delivery from drug–interpolyelectrolyte complexes exhibited a remarkable robustness toward changes in pH of receptor media [60,61].

#### 3.2.2. Liposomes

Liposomes (Figure 1e) are well-established, phospholipid-based, biocompatible vesicle systems that form the basis for several FDA-approved therapeutic products [62]. In recent years, the use of liposomes in diagnostic and theranostic applications has increased, and a wide variety of diagnostic and therapeutic agents have been encapsulated in liposomes for this purpose [63]. For example, Cai et al. demonstrated the integration of an AIE fluorogen in a liposome for image-guided drug delivery, exhibiting bright red fluorescence along with in vivo antitumor efficacy after white light illumination (photodynamic therapy) [64]. Wang et al. developed a liposome-based fluorescent “turn-on” nanosensor to map the spatial expression of the biomarker Mucin 1 in cancer cells, functionalizing the liposomes with a Cy3-labeled aptamer-binding Mucin 1 [65]. To facilitate the translation from bench to bedside, several research groups have focused on the encapsulation of the FDA- and EMA-approved indocyanine green into liposomes, and several different therapeutic or theranostic applications have been demonstrated, such as phototherapy in mouse breast tumor [66], synergic combination of chemotherapy and hyperthermia for cancer phototherapy [67], and light-triggered drug release [68].

#### 3.2.3. Quatsomes

Quatsomes (QSs, Figure 1e) are nanovesicles composed of quaternary ammonium surfactants and sterols, which form small unilamellar vesicles in the size range of 30–100 nm, exhibiting a high structural homogeneity and a stable size and morphology over years for many formulations [69]. QSs have been used as scaffolds for the nano-structuration of lipophilic fluorescent dyes (such as carbocyanines DiI, DiD, and DiR) in aqueous media, obtaining optimal colloidal stability and photostability [70] and excellent performance for in vivo bioimaging as demonstrated in mice [71]. QSs containing a pair of carbocyanine dyes undergoing FRET have been shown to exhibit very high brightness (B = 7 × 10^7^ M^−1^ cm^−1^, 120-fold brighter than QDot 605), and a particle-to-particle variation in brightness generally below 10% [72,73], both critical features for bioimaging probes. QSs can be easily functionalized through the cholesterol moiety [74], and the functionalization of DiI-loaded QSs with DNA/Alexa Fluor 647 probes have been shown to enable the highly selective, ratiometric detection of clinically relevant microRNAs through FRET, with sensitivity in the low nanomolar range [75].

#### 3.2.4. Lipid Nanoparticles

In contrast to nanovesicles, lipid nanoparticles have a hydrophobic core (Figure 1g), which can facilitate high dye loading and increased brightness. Lipid nanoparticles comprise both solid lipid particles (SLNs), which mainly contain lipids in the solid state at room temperature, and nanostructured lipid carriers (NLCs), which are nanoemulsions formed of both solid and liquid lipids [9]. In an early demonstration, Texier et al. confirmed the loading of lipophilic carbocyanines DiD and DiR in lipid nanodroplets, obtaining fluorescent probes with high colloidal stability (over one year) and improved fluorescence quantum yields [76]. The avoidance of aggregation-caused quenching at increased dye loadings (up to 8% *w*/*w*) was achieved by replacing the small hydrophilic counterion of DiI with the bulky hydrophobic counterion tetraphenylborate, yielding a brightness as high as B = 8 × 10^7^ M^−1^ cm^−1^ [77]. In vivo imaging of nanocarrier integrity upon intravenous injection in mice was demonstrated using FRET-presenting nanoemulsions with high loadings of donor (Cy5.5) and acceptor (Cy7.5) dyes, monitoring donor and acceptor emission upon donor excitation in a whole animal imaging setup. Large differences of nanocarrier integrity in blood circulation and tumor were found [78].

#### 3.2.5. Polymeric Nanoparticles

Polymeric nanoparticles (Figure 1a) with well-controlled surface properties can show a remarkable stability in biological environments. To achieve superior brightness, many efforts have been dedicated to load large amounts of dyes into polymer NPs in the absence of ACQ, either by means of AIE [79], dye modification with bulky side groups [80], or bulky counterions [81]. Klymchenko et al. also developed ultrabright nanoantennas that enable single-molecule detection at illumination powers of only 1–10 mW cm^−2^ (>10,000-fold lower than typically required in single-molecule measurements) [82]. In addition, the highly sensitive, ratiometric detection of microRNAs under point-of-care conditions was demonstrated [83].

#### 3.2.6. Polymeric Micelles

Polymeric micelles (Figure 1b) are colloidal aggregates, ranging in size between 10 and 200 nm, resulting from the self-assembling of amphiphilic polymers when in aqueous solution. Thus, micelles show an inner lipophilic core, made by the hydrophobic moiety of the polymer, surrounded by a hydrophilic shell. The formation of micelles occurs when the polymer concentration is above a critical value, known as critical micelle concentration (CMC), which is specific for each polymer. Di-block copolymers, such as poly(ethylene glycol)-poly(ε-caprolactone) (PEG-PCL) and poly(ethylene glycol)-poly(lactide-co-glycolide) (PEG-PLGA), tri-block, such as poloxamers, as well as ionic copolymers are among those commonly used in the pharmaceutical field for micelle preparation [84]. Several methods are available for micelle preparation and loading of the hydrophobic compounds: the direct dissolution method, dialysis, emulsion with solvent (or co-solvent) evaporation, and film hydration [85]. Regardless of the preparation method, micelles are thermodynamically stable, highly versatile, suitable for lyophilization and sterilization, and, therefore, extensively studied as drug delivery platforms, especially for oral [86], topical (dermal and transdermal) [87], and mucosal (i.e., ocular, nasal, or pulmonary) administration [88]. Polymeric micelles formed by a di-block copolymer of PEG and a poly(methyl methacrylate) derivative covalently conjugated to functionalized indocyanine green are currently explored in clinical trials (phase I/II) for their use in fluorescence-guided surgery of four different solid tumor types, including breast cancer, as well as head and neck squamous cell carcinoma. The administered doses were well tolerated and enabled the detection of occult lesions (such as satellite metastases and second primary tumors) missed by standard of care surgery [89].

#### 3.2.7. Polymersomes

Polymersomes are nanovesicles resembling liposomes (see Section 3.2.2), which are composed of amphiphilic block copolymers instead of phospholipids to form the membrane [90]. Particularly, di-block and tri-block copolymers (see Section 3.2.6), such as PEG-PLGA, PEG-PCL, and poloxamers, are commonly selected with the aim of preparing highly stable nanosystems for drug delivery and diagnostic purposes [91]. 

Polymersome preparation has been shown using different approaches such as thin film hydration, direct injection, double emulsion, and microfluidics [91,92]. Apart from the choice of the polymer, the selected preparation method influences the nanovesicles characteristics in terms of morphology and size [93].

Fluorescent polymersomes have been proposed for their use in bioimaging and theranostics, for example BODIPY-loaded polymersomes for photodynamic and photothermal therapy in cancer [94] and polymersomes containing fluorescein isothiocyanate derivatives for drug delivery [95].

In addition, the preparation and characterization of fluorescent polymersomes containing tetraphenylethylene (TPE) were described, showing interesting aggregation induced emission (AIE) characteristics [96].

## 4. Practical Guide for Manufacturing FONs

Choosing the best fabrication technique for FONs depends on the selected material and the target in terms of the NP’s physicochemical properties (such as size, polydispersity, ζ-potential, stability, etc.) as well as the production method (i.e., sample concentration, yield, volume/amount per day, etc.). This section will describe an overview of the production methods for FONs.

FONs synthesis techniques are essentially categorized as bottom–up approaches, wherein molecules assemble into larger stable nanoparticles, and top–down methods, such as the milling of bulk materials via ultrasound or laser ablation.

**Figure 5:**
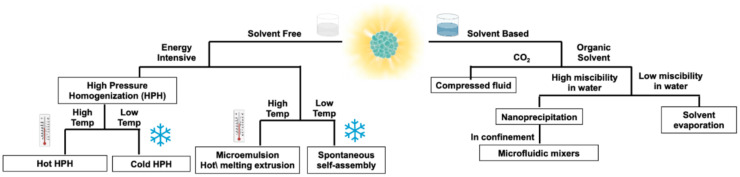
Main methods for FONs production.

Figure 5 illustrates different available techniques; FONs components (fluorophores, polymers, monomers, lipids, etc.) and the hydrophilicity of the cargo, should be considered when selecting the proper FONs preparation method.

### 4.1. Solvent-Free Fabrication Techniques

#### 4.1.1. High Pressure Homogenization

High pressure homogenization (HPH) is well suited for lipid nanoparticle production. It consists of creating an emulsion with the molten lipids, water, and surfactants using a high-speed stirrer and subsequently pushing the solution through small orifices using high pressures (>100 bar). The high velocities reached by the fluid are accompanied by turbulence, high shear forces, and cavitation, which allows for the formation of FONs even at high concentrations. This method exists in two different protocols depending on the temperature used in the process: hot HPH or cold HPH [97]. Craparo et al. combined this method with a solvent evaporation technique to fabricate FONs of less than 150 nm with rhodamine B-conjugated block copolymers [98].

#### 4.1.2. Spontaneous Self-Assembly in Aqueous Media

Hou et al. reported the spontaneous self-assembly of water dispersible FONs for cyanide detection. Through a series of seven chemical reactions, they successfully synthesized a dilactosyl–dicyanovinyl-functionalized tetraphenylethene (TPELC), which spontaneously formed NPs in water when the CMC was reached [99]. In other cases, self-assembly is driven by the self-polymerization of organic compounds, as demonstrated by Shi et al. In this study, 50 nm FONs were synthesized by the self-polymerization of a polydopamine (PDA) complex with polyethyleneimine (PEI) copolymers in deionized water [100]. This method has the advantage of being a one-step process in mild conditions without the need for harmful solvents and reagents. However, the compounds capable of such self-assembly in aqueous media are limited and some only exhibit this behavior when organic solvents are present, as discussed by Zhao et al. [101]. Here, polydopamine FONs were synthesized by self-polymerization in ethanol at alkaline pH. The intensity and peak position of the emission spectra were tuned by dispersing the FONs in various solvents, including water.

In a similar fashion, the formation of PECs is a spontaneous self-assembly and a safe and green process. In fact, the interaction of anionic or cationic PE with oppositely charged drug/dyes yields a high proportion of counterionic condensation and stable aqueous colloidal dispersions [102,103]. In some cases, the complementary addition of an inorganic counterion contributes to the required degree of dispersibility. For instance, the interaction of the linear polymethacrylate Eudragit^®^ E with organic acids yields stable aqueous dispersions when an inorganic anion (e.g., Cl^−^) is incorporated into the system [57,104]. Water removal by evaporation, spray-drying, or freeze-drying resulted in solid-state PECs, which are also characterized by an ionic interaction between the PEs and the drug [105]. In many cases, solid PECs spontaneously revert to the original nanosized colloidal dispersion upon contact with an aqueous medium.

#### 4.1.3. Microemulsion

The microemulsion method can be used to fabricate polymeric and lipid nanoparticles. Oil-in-water (o/w) or water-in-oil (w/o) microemulsions are prepared by mixing two non-miscible liquids in the presence of surfactants (i.e., surfactant(s)/co-surfactant(s) mixture) until a stable dispersion is formed. Organic nanoparticles can be obtained in w/o microemulsions by direct precipitation in the aqueous droplets. The nucleation and growth of nanoparticles in microemulsions is a complex process and theoretical models were adapted, notably to account for the impact of surfactants on NP stabilization [106]. The water/surfactant ratio is very important in controlling the phase separation as well as submitting the mixture to ultrasounds to provide sufficient energy in the system [107]. O/w is limited to the production of a few types of polymeric NPs with a method called microemulsion polymerization, wherein hydrophobic monomers are polymerized by the addition of an oil-soluble initiator [108,109]. To produce solid lipid nanoparticles, a two-step process can be used wherein a first hot emulsion is made of molten lipids, surfactants, and water, and then this microemulsion is added to cold water and stirred until precipitation of the lipid phase [110]. However, this method requires a large amount of surfactant to stabilize the microemulsion, which is currently a challenge for most applications [111].

#### 4.1.4. Hot Melt Extrusion

Hot melt extrusion (HME) involves the processing of PE or polymeric materials at temperatures above their melting point or glass transition to achieve ionic interaction or molecular-level mixing with an active compound. As with microemulsions, no solvent is needed for the fabrication process [51,97,112]. HME has been traditionally used for developing solid particles such as granules and microgranules, while recent advances have been explored by coupling HME with additional technologies. In fact, semi-solid nanostructured lipid carriers were obtained by using HME coupled with probe sonication [113]. Moreover, HME combined with high-pressure homogenization allowed one to obtain nanocrystal solid dispersions [114]. In addition, a one-step nano-extrusion process for transferring aqueous nano-suspensions into solid formulations directly in the liquid phase has also been reported. In this case, nano-suspensions were fed into molten polymers and excess water was eliminated via evaporation [115].

### 4.2. Solvent-Based Fabrication Techniques

#### 4.2.1. Nanoprecipitation

This method essentially relies on the fast mixing of two miscible phases, an aqueous phase, and an organic phase (the latter containing the fluorophores, the polymers, or the lipids that are not soluble in the aqueous phase). The rapid dilution of the organic solution into the non-solvent leads to the self-assembly of the fluorophores, polymers, lipids, and/or surfactant units. NPs constituted by small fluorophores can be obtained in this way, as well as nanoemulsions of polymers or lipids, wherein each NP can encapsulate the fluorophores. Typically, the starting material is dissolved in an organic solvent, and then a small amount of this solution is injected into a larger volume of water or aqueous buffer (often containing surfactants) [116]. Extrusion, pushing the nanoemulsions through membranes with decreasing pore sizes, and sonication are methods often applied to reduce and refine the particle size distribution of FONs [117,118,119]. Many parameters can be changed to obtain FONs with the desired physicochemical properties. In a recent study, Kaur et al. demonstrated the effect of the organic solvent, as well as the concentration of the organic compound, temperature, and pH on the size and polydispersity index of benzothiazole-based FONs [120]. The volume ratio of the aqueous phase to the organic phase exerts an important impact on the final size distribution of the nanodispersion. Injecting a large amount of organic phase with respect to the aqueous phase leads to the formation of larger NPs and agglomeration [120].

#### 4.2.2. Solvent Evaporation

Like the nanoprecipitation technique, FONs are prepared by dissolving polymers or lipids and encapsulating the fluorophores in an organic solvent before injecting the solution in water or an aqueous buffer. In contrast to the nanoprecipitation method, the solvent is completely immiscible or only partially miscible with water. After the emulsification of the organic phase into the aqueous phase under continuous stirring, the solvent is evaporated, and the nanoparticles are purified [97].

#### 4.2.3. Compressed Fluid (CF)-Based Preparation

CF-based technologies present several advantageous features for nanoparticle processing, such as high throughput in the production of high quality, homogeneous nanoparticles, adequacy for industrial scale production, and compliance with the principles of green chemistry [121,122]. Various CF-based nanoparticle production technologies have been developed [123,124], giving rise to a large variety of nanoparticle systems with differentiated characteristics. Generally, the CF can act as a solvent, anti-solvent, or co-solvent, depending on the solubility of the solutes in the CF [123].

Carbon dioxide (CO_2_) is the most widely used gas in CF technologies because it is inexpensive, non-toxic, non-flammable, non-corrosive, and easily separated from the products. Due to its low critical temperature of 31.1 °C, processes (also known as supercritical fluid processes) can be carried out at mild temperatures, avoiding the thermal degradation of the labile compounds. Since several CF-based methodologies reduce the amount of organic solvent required in nanoparticle preparation, CO_2_ has become a “green substitute” to organic solvents, complying with several principles of green chemistry, such as pollution prevention, lower toxicity, and the use of an abundantly available resource [123,124,125].

Several CF-assisted technologies have been optimized for the production of nanovesicles [126,127]. The preparation of fluorescent lipid nanovesicles, in particular, was demonstrated using the CO_2_-based method named “Depressurization of an Expanded Liquid Organic Solution” (DELOS) [74,128], producing a clearly superior vesicle-to-vesicle composition homogeneity compared to thin film hydration-based production [129], which is not necessarily evident in bulk measurements [70]. The high vesicle-to-vesicle homogeneity was confirmed for FRET-presenting nanovesicles, showing brightness and FRET ratio variations generally less than 10% [73].

#### 4.2.4. Microfluidic Mixers

The state-of-the-art process for the synthesis of organic NPs in microfluidics is by solvent–antisolvent nanoprecipitation. Mixing in microfluidics is usually slow as the flow is laminar and mixing time is generally controlled by diffusion only. However, strategies have been developed to improve the mixing efficiency while profiting from the benefits of laminar flow and confinement [130]. These features allow for the precise tuning of the nanoparticle’s size, while ensuring a narrow-size distribution and high reproducibility, as demonstrated by Hoang et al. [131]. Using a microfluidic chip for flash precipitation of two fluorophores led to the production of small-sized photocrosslinkable FONs with high reproducibility as compared to those obtained by manual solvent injection and vortex stirring methods [131].

**Figure 6:**
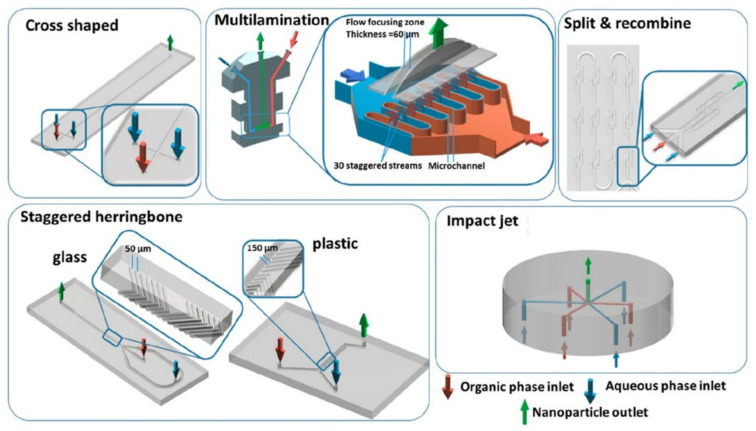
Overview of microfluidic mixers used for nanoparticle preparation. (Adapted with permission from [132]. Copyright 2022 American Chemical Society).

Whether the fluorophores are encapsulated or conjugated within the nanoparticles, the size will impact their fluorescent properties. Chen et al. studied many types of microfluidic mixers (see Figure 6) to fabricate polymeric NPs in PLGA or PMMA encapsulating rhodamine-B based fluorophores, and compared their performances with conventional bulk nanoprecipitation methods [132]. Their findings show that most of the nanoformulations prepared in microfluidics have similar or better qualities (i.e., size and PDI (polydispersity index)) than those prepared in bulk, and have the advantages to be produced continuously, without the need of an extra step of size adjustment (e.g., sonication or extrusion). The versatility of this approach and the potential easy up-scaling of the production [133,134] makes this method a powerful tool for future FONs developments. Additionally, high-pressure microfluidic systems have been developed, and the preparation of FONs using a supercritical non-solvent process has been shown [135].

## 5. Optical Characterization of Fluorescent FONs: From Spectroscopy to Fluorescence Microscopy

The ease in the optical spectroscopic characterization of FONs strongly depends on whether they scatter light employed in the measurements. Light scattering depends on the size of the NPs with respect to the relevant wavelengths: if NPs are much smaller than the wavelength of the light, scattering will be negligible; if the size of the NPs is comparable or larger than the wavelength, light typically is strongly scattered, resulting in unreliable spectra [136]. A good indication of scattering can be observed if the UV–vis absorption spectrum exhibits a non-straight and/or non-zero baseline. Of course, performing dynamic light scattering (DLS) characterization can provide more detailed information on scattering, including the size and polydispersity of the NPs [137].

### 5.1. Good Practices for the Spectroscopic Characterization of FONs

Optical spectroscopic measurements on insignificantly scattering NP suspensions are straightforward, since they can be conducted by adopting the same good practices as for solution measurements. On the contrary, optical spectroscopic analysis on highly scattering suspensions is much harder and only limited to accurately investigating a few properties. A brief summary of the good practices for spectroscopic characterization is reported in Table 1.

The absorption spectra of highly scattering samples cannot be measured in transmission mode since the spectrum would be strongly affected by scattering and the apparent absorbance would be strongly underestimated [140]. The best way to correctly measure the absorption of a highly scattering sample is by using an integrating sphere accessory in the spectrometer [140]. Measuring the fluorescence spectra of scattering suspensions requires several precautions, since both the incident light and the emitted light can be scattered by the sample. Scattered light appears in the emission spectrum not only at its real frequency (or higher-order diffraction from the grating) but can appear at practically any wavelength in the form of stray light (light that passes through the monochromator in addition to the desired wavelength) [139,141]. The use of double-monochromator fluorometers helps in reducing scattering and stray-light contributions but, even so, appropriate optical filters in the emission and/or excitation path are needed [139]. Fluorescence quantum yields of the scattering samples can be estimated using an integrating sphere accessory in the fluorometer [139,142]. Fluorescence lifetimes can be quite safely estimated for scattering samples, preferentially in the front-face configuration or placing the sample into a short-path cuvette [141].

### 5.2. General Remarks for Retrieving Information from Spectroscopic Investigations of NPs

For both scattering and non-scattering suspension samples, contrary to solutions, the UV–vis absorbance does not necessarily have to follow the Lambert–Beer law: when preparing more dilute (or more concentrated) suspensions, the size and shape of the NPs themselves can change (accordingly to the type of NPs), and this can bring about concomitant changes (shifts, narrowing/broadening) of the absorption spectrum that do not comply with the Lambert–Beer law [143].

Concerning emission spectra, Kasha’s rule is typically adhered to when the NPs in the suspensions are highly homogeneous (one size, one shape, i.e., they are alike), as in the case of isolated fluorophores: the emission spectrum is independent of the excitation wavelength and, analogously, the excitation spectrum is independent of the emission wavelength and superimposable with the absorption spectrum. However, when the NP suspension is inhomogeneous (polydisperse, or composed of NPs with varying shape or density), the specific excitation wavelength can preferentially photoselect a subset of NPs out of the inhomogeneous ensemble, so that the emission will preferentially stem from that specific subset [144].

In NP suspensions, even if the global concentration of fluorophores is low, a high local concentration of fluorophores can be achieved within each NP, a situation that should be considered during data analysis. In this case, fluorophores can easily interact and, depending on their distances, exhibit FRET [72] or excitonic effects [145,146,147,148]. FRET occurs when interchromophore distances are on the order of 20–100 Å and consist of the exchange of excitation energy from one fluorophore to another, provided that the emission spectrum of one fluorophore spectrally overlaps the absorption spectrum of the other [149]. If energy transfer is highly efficient, the observed emission shall stem from the fluorophores having the first electronic excited state at the lowest energy. Excitonic effects are observed at shorter interchromophoric distances, typically on the order of 4–20 Å, when aggregates are formed [150]. The absorption and fluorescence spectra of aggregates can be quite similar or even very different from the corresponding spectra of the isolated fluorophore, depending on the strength of interfluorophore interactions in the NP. For strong intermolecular interactions, the spectra are typically different from those of the isolated fluorophore, showing the typical characteristics of aggregation (J- or H-aggregates, according to the specific molecular packing) [37].

### 5.3. Fluorescence Anisotropy: An Important Tool for Non-Scattering Samples

Important information on emissive objects can be obtained by fluorescence anisotropy, defined as (1) [139]:

(1) $$r=\frac{I_{∥}-I_{⊥}}{I_{∥}+2I_{⊥}}$$

where $I_{∥}$ and $I_{⊥}$ are the fluorescence intensities measured when exciting with vertically polarized light and detecting emission through a polarizer being aligned parallel or perpendicular, respectively, with respect to the original polarization direction. Fluorescence anisotropy is a measure of how strongly the emitted light is polarized after polarized excitation. Its value strongly depends on how fast the emitting objects rotate in the solution/suspension during their excited-state lifetime. If the rotation is much faster than the de-excitation rate, the emission is completely depolarized (r=0); if the rotation is much slower than the decay rate, the emission is strongly polarized (r=0.4 is the maximum value in solution/suspension); in intermediate cases, when the rates of rotation and of emission decay are comparable, fluorescence anisotropy assumes intermediate values [139].

In common solvents, the emission of small fluorophores is usually (almost) completely depolarized (only in viscous solvents the rotational correlation time of small molecules becomes long enough to observe significantly polarized emission). The case of NPs is different: they are “large” objects, whose typical rotational correlation times in common liquids (such as water) can be in the order of ns (or fractions of ns), comparable with typical fluorescence lifetimes [151]. This means that NP suspensions typically give rise to a partially polarized emission (r≠0). In the absence of any other depolarization cause other than the NP’s rotation, the Perrin equation applies (2) [139]:

(2) $$r=\frac{r_{0}}{1+\frac{\tau }{\theta }}$$

where $r_{0}$ is the fundamental anisotropy (the one in the absence of rotation, such as in highly viscous matrices or vitrified solvents), τ is fluorescence lifetime, θ is the rotational correlation time (only spherical objects have a single rotational correlation time; for more complex shapes, multiple correlation times should be considered). For a given solvent and temperature (density η, temperature T), the rotational correlation time only depends on the volume (V) of the rotating object (3) [139]:

(3) $$\theta =\frac{\eta V}{RT}$$

therefore, the measure of fluorescence anisotropy (together with the fundamental anisotropy $r_{0}$) allows for the estimation of the size (volume) of the emitting object.

Quite precise information can be obtained, not only on the size, but also on the shape of the NP by measuring the time-resolved fluorescence anisotropy decay. For a spherical object, the anisotropy decay follows a simple mono-exponential law (4):

(4) $$r\left(t\right)=r_{0}\exp\left(-t/\theta \right)$$

but an object having a generic shape rotates at different rates around different axes, so a multi-exponential anisotropy decay is expected. The interested reader is referred to more technical readings for further details [139,152].

The measure of fluorescence anisotropy is, however, too sensitive to scattering, so correctly measuring the anisotropy of turbid samples is practically impossible, even when filters are used. Both incident and emitted light can be scattered while passing through the cuvette, and any scattering event is a source of depolarization. Most of the time, the emission of turbid samples is completely depolarized by scattering. The only way to reduce this effect is by using cuvettes with very short path lengths, so as to reduce the scattering probability [152].

### 5.4. FONs for Bioimaging: Key Spectroscopic Parameters to Be Monitored

As mentioned above, FONs are commonly developed to work as efficient fluorescent probes for bioimaging. For this application, brightness is a highly important figure of merit: for one-photon excitation processes, brightness is defined as the product of the molar extinction coefficient and the fluorescence quantum yield [153]. For two-photon excitation, as is relevant for two-photon microscopy, a similar brightness can be defined, wherein the molar extinction coefficient is substituted with the two-photon absorption cross section. Fluorescent nanoparticles are engineered to maximize their brightness, i.e., increasing the extinction coefficient while maintaining a high quantum yield. The principle for achieving high brightness in FONs relies on the confinement of a large number of fluorophores (typically hundreds or thousands) in a nanosized structure. As such, the brightness of the particle can be much higher compared to that of the single fluorophore, thanks to the much larger absorption coefficient of the particle ($\varepsilon_{p}$) according to Equation (5):

(5) $$\varepsilon_{p}=n\varepsilon_{f}$$

where $\varepsilon_{f}$ is the absorption coefficient of the single fluorophore and *n* is the number of fluorophores in the nanoparticle.

The increase in the molar absorption coefficient obtained by increasing the local concentration of dyes in the nanoparticle has a positive effect in increasing the brightness. However, the fluorescence quantum yield should also be monitored in order to avoid aggregation quenching effects [154]. In fact, strong intermolecular interactions (mostly π-π stacking) may cause aggregation quenching, resulting in a detrimental effect on the brightness. Different strategies have been developed to prevent aggregation-caused quenching. One approach is introducing bulky groups [29,30,31] or polymer chains [33] into the fluorophore’s molecular structure, to prevent π-π stacking. Other strategies rely on, e.g., the co-assembly of chromophores and molecular barriers [155] or the exploitation of the counterion effect [156]. Additionally, AIE can be exploited to obtain highly fluorescent NPs [34], but the molecules constituting the NP have to be AIE dyes, which are relatively limited in number and variety [36].

### 5.5. Fluorescence Microscopy

FONs are commonly exploited as fluorescent probes in different fluorescence imaging techniques. Several imaging techniques have been developed in which fluorescence is the signal detected that creates an image contrast. Table 2 reports different fluorescence imaging techniques, characterized by different working principles, and a variety of experimental setups: (i) linear techniques are the ones in which the excitation of the sample is promoted by the absorption of a single photon (linear absorption), while (ii) in non-linear techniques multiple photons (usually two) are absorbed simultaneously to excite the sample. In both cases, independent of which process is responsible for excitation, fluorescence is detected.

Among the different imaging techniques, multiphoton microscopy (MPM) is very useful to follow the fate of FONs in biological tissues, opening the possibility to monitor drug delivery over time. A multiphoton microscope collects the light emitted by dyes excited via two-photon absorption (2PA). In 2PA, a molecule is promoted to the excited state via the simultaneous absorption of two photons, a non-linear optical process [166]. Compared to the linear (one photon) process, less energetic photons are used in 2PA, typically in the NIR. Apart from reducing the photodamage caused by UV and visible light, NIR light falls in the transparency window of biological tissues, allowing for relatively deep imaging. Moreover, the 2PA probability is proportional to the square of the light intensity: the 2PA-induced fluorescence is confined in the small (yet diffraction-limited) volume defined by the laser focus (the voxel), where the light intensity is large enough to trigger the process, making two-photon microscopy (2PM) an imaging technique with intrinsic 3D resolution. The 2PM setup can also be exploited to collect second-harmonic generated (SHG) light, produced at twice the frequency of the laser irradiation source thanks to a second-order non-linear interaction between the incoming electromagnetic field and the sample. The SHG signal is only generated in locally non-centrosymmetric media, so that signals from dyes inserted in cell membranes or from specific asymmetric structures (e.g., collagen fibers) can be selectively detected.

## 6. Computational and Theoretical Approaches for Modeling FONs

Computer simulations represent powerful tools that can facilitate the design and application of NPs and FONs, providing details at the molecular level with in-depth information on structural, dynamic, and opto-electronic properties. Moreover, they can assist in the rational design of new formulations and optimization of properties, in addition to improving in vivo activities [167]. In general, the modeling of organic NPs can be used for an improved understanding of critical aspects such as: self-assembly and formation of nanoparticles; structural and dynamic characteristics of the NPs/aggregates formed; interaction between the drug/dye and nanoparticles; and the design optimization of the targeting ability. The simulation framework could also enable the investigation of specific environmental factors, such as pH, temperature, counterions, ionic strength, and the stability and integrity of the nanoparticulate system. 

In the field of nanomedicines, molecular simulations have been widely employed, especially in the framework of atomistic molecular dynamics. Micellar NPs as drug nanocarriers have been recently reviewed, focusing both on the computational design and the optimization of micellar nanomedicines and their nanomedicine activity, including adsorption, enzymatic activity, multivalent blocking of active sites, and destabilization of viruses and fibrils [168], as well as biological barriers [169]. For applications in drug and gene delivery, NP-nucleic acid systems have been investigated at different levels with molecular simulations, as reviewed by Nash et al. [170]. However, among organic NPs, most of the simulations relate to drug-delivery rather than FONs. Still, the ability to simulate and predict the behavior and properties of NPs in different environments may translate into similar challenges for all NPs, fluorescent or not. A comprehensive simulation of FONs that could describe their morphology and dynamic properties while at the same time predict the optical properties of the fluorescent dye is quite challenging due, in part, to its large size and the inherent complexity of the system and environment.

As a key point in materials and molecular modeling, the level of theory (accuracy) of the simulation must be adjusted, depending on the problem depth and resolution needed, for the available computational resources and on the system size. Based on its computational cost, each modeling technique allows one to simulate a typical range of time and/or space scales. For example, the simulation of a whole organic nanoparticle could be achieved by employing classical potentials, either all-atom (AA) or coarse-grained (CG), usually in the context of molecular dynamics. On the other hand, the simulation of electronic properties requires methods that can describe electronic degrees of freedom; therefore, it is computationally demanding and imposes limits on the simulation size. 

In Table 3, some of the most common techniques and their applications in the field of organic NPs are summarized, including non-fluorescent organic NPs for drug delivery, as they share some of the physicochemical processes and the modeling techniques with FONs.

In addition, studies on molecular (atomistic) modeling schemes are only briefly mentioned, while Monte-Carlo simulations or meso-scale and discrete-to-continuum-like approaches such as dissipative particle dynamics (DPDs) are not discussed. For these approaches in the context of nanomedicine and NPs for drug delivery, the reader is invited to consult some of the recent reviews [183,184]. In addition, the related field of fluorescent membrane probes has been significantly investigated by simulations, as recently reviewed [185].

As highlighted in Section 2 and Section 3, FONs can be constructed with fluorescent building blocks, or dyes that can be loaded into the NPs. In simulating systems such as dye-loaded NPs, treating the organic nanoparticle and the dye with the same level of theory is practically unfeasible. The former is typically approached by classical potentials (AA or CG, see Table 3), while as the dye is responsible for the optical properties that we aim to interrogate, density functional theory (DFT) techniques are more suitable for this task (see [172] and references in Table 3). These simulation schemes are often referred to as hybrid or multi-scale, and could also be implemented more specifically in the form of QMs/MMs (quantum mechanics/molecular mechanics), as achieved in the context of molecules with optical responses embedded in biological matrices [186]. The possibility to explore dynamic information is usually conducted with AA or CG classical potentials, in the frame of molecular dynamics (MDs) simulations, wherein the (classical) equations of motion are employed to describe the evolution of “particles” (atoms, molecules, etc.) through their position and velocities, although, in principle, forces can be computed at any desired level of theory. Through an MDs trajectory, temperature and pressure can be set, and then a realistic sampling of the system’s degrees of freedom could be approached. 

Among the works that aim to deal with both scales, Lescos et al. provided a key to the relation between the structural organization of the dyes in FONs and the second-order nonlinear optical properties [172]. The molecular dynamics simulations of the FONs’ formation in water were combined with quantum chemical calculations, describing the molecular aggregation process, the molecular orientation of the dipolar dyes within the nanoparticles, and the dynamic behavior of their non-linear optical properties. To evaluate the optical response, in spite of the large number of atoms, a simplified version of TDDFT (called s-TDDFT) was employed. 

Quatsomes (QSs) were also investigated by simulations in several joint-experimental works, mostly by selecting a QSs bilayer as model system and focusing on the dynamic properties of the dyes in such membranes as key elements to evaluate brightness and energy transfer [70,73]. CG schemes for describing the self-assembly of cationic surfactant into micelles was proposed in reference [182] employing Martini CG Force-Field [179] in the implicit vs. explicit solvent implementation, obtaining different average micelle sizes while still accounting for the physics of these classes of organic NPs. When employing all-atom approaches, the vesicles employed as a model system are generally smaller than the realistic ones. Nevertheless, AA–MDs simulations are generally sufficient to assist and rationalize the observed experimental behavior [178].

We highlight that, as a general strategy, to describe complex systems such as FONs and their properties, a hybrid or multiscale approach is desirable, as the treatment of the entire system at the highest possible level of theory is not feasible. However, it is expected that advances in the computational power will improve the description of these systems, with more realistic and full dynamic information, so as to provide important support and complement ever-evolving experimental approaches in FON formulations, and toward their structural and functional optimization.

## 7. Applications

There are manifold applications for fluorescent multifunctional nanoparticles in a variety of fields, which include: sensing [187], imaging [188], diagnostics [189,190], and drug delivery [12,191] (some examples are reported in Table 4). While there are numerous reviews that provide a good overview of such applications [192,193,194], in this review, we will provide a few highlights of the latest applications and uses of FONs.

### 7.1. Sensing

One of the main advantages of FONs is the integration of unique fluorophores with specific materials, which can be used as sensors for different chemicals [204,205], proteins [206], and other materials [207,208]. Specific examples are FONs as metabolic sensors: these sensors afford approaches to tackle a major challenge in monitoring the metabolic changes of the microenvironment around cells and tissues in situ (e.g., oxygen, reactive oxygen species, and pH). Such information can be used to assess the metabolic state of the tissue, the phenotypic state of the cell, and to characterize drug effects on cells [209]. FONs as hormone sensors: due to their modularity and specificity, FONs can be modified and used to identify specific proteins and hormones either in vitro or in vivo [206,210,211]. Such FON sensors were applied to identify the secretion of neurotransmitters (e.g., dopamine [212], GABA [213], etc.) and other important hormones, such as insulin [214] and triiodothyronine [215]. These FONs offer several advantages in addition to standard electrochemical sensing methods, including high sensitivity, spatial information on the compound distribution, high specificity, and facilitating the ability to sense multiple compounds simultaneously using different FONs at different wavelengths.

FONs sensors can be integrated within advanced in vitro platforms. In recent years, new in vitro tools that better mimic human physiology were established. These platforms include 3D platforms, Organs-on-a-Chip (OoCs), microphysiological systems (MPSs) [216,217], and organoid platforms [218]. These systems enable one to induce flow, to use human tissues, to examine organ–organ interactions, and to mimic complex 3D structures and specific microenvironments. Due to their unique structure, embedding sensors in such platforms is challenging. However, the use of FONs enables the embedding of a variety of sensors (as mentioned above) as part of Organs-on-a-Chip [209,219], organoids [220,221], and as parts of complex 3D systems [222] using 3D printing or other techniques.

### 7.2. Bioimaging and Diagnostics

The ability to specifically target and identify chemicals, DNA, RNA, and proteins with spatial precision enables the use of FONs for bioimaging and spatial diagnostics. An excellent example for such use was recently demonstrated by Blau et al. [223], who designed a “turn-on” probe, which included fluorescently tagged activatable cell-penetrating peptides (ACPPs) that can elucidate tumor boundaries during surgery, enabling clinicians to visualize the area required for removal during the process, thereby increasing the success of the operation [223]. This work provides an excellent example of the strength of FONs for both bioimaging and diagnosis. Another example includes the identification of ions of Al^+3^ in bacterial samples [193], ionic ratios [205], and tumors [224].

### 7.3. Therapeutics

There are many examples of the use of FONs in therapeutics. It was shown that NPs can both enhance the therapeutic effect of specific drugs and can be used for therapeutic purposes themselves [191]. It is important to note that some of these NPs are FDA-approved and are currently in clinical use [195,225,226]. Particularly, in this review, we refer to the term “targeted therapeutics” for carrier systems delivering drugs to a specific location, cell, or organ, based on the interactions with specific receptors, via surface functional groups or properties of FONs. On the other hand, we will refer to “drug delivery” as the use of NPs to carry drugs across biological barriers and toward tissues which could otherwise not be attained.

An example in the field of targeted therapeutics is the use of shear-activated PLGA nanoparticles, which release the tissue plasminogen activator (i.e., a protein involved in the disruption of blood clots) only in areas where the blood vessels are clogged [198]. Another study addressed the use of pegylated liposomes to target neurons in the tumor microenvironment, thus reducing breast cancer progression and metastases [196]. Several studies can be found in the literature addressing the use of fluorescent and fluorophore-loaded nano- and micro- carriers for systemic (intravenous) and topical (dermal delivery) administration. A classic example is the use of FONs for crossing the blood–brain barrier (BBB) [227,228,229], which is known to be a major challenge and the main limiting factor for drug delivery to the brain [230]. The use of such FONs as a platform for drug delivery enables one to monitor how the FONs cross the BBB and arrive to their specific target inside the brain [180,231,232].

Lunardi and co-authors proposed theranostic agents of nano- and micro-particles poly(lactic-co-glycolic) acid (PLGA) loaded with cresyl violet, a basic dye (λ_abs_ 540 nm; λ_em_ 632 nm) [199]. Once prepared and characterized, the NPs (approx. average size 350 nm; negatively charged) and micro-particles (approx. average size 950 nm and 5 μm; negatively charged) were intravenously injected in anesthetized mice. The animals were then euthanized and dissected to isolate the spleen, lungs, heart, kidney, and liver. MPM allowed for the observation of PLGA particle distribution within the different organs, thus confirming the capability of the developed FONs to easily reach different targets [199]. Theranostics appears particularly relevant, especially in the case of tumors. Therefore, a miniaturized two-photon endoscope designed for the simultaneous delivery of anticancer doxorubicin and real-time bioimaging of the tumor has recently been proposed [233]. Doxorubicin is an anticancer drug approved by the FDA almost forty years ago [195] and available on the market also as liposomal formulation for intravenous administration [195]. Doxorubicin has intrinsic fluorescent properties (λ_abs_ 470 nm; λ_em_ 595 nm), and is thus a very useful tool for both researchers and clinicians [234].

MPM not only provides a non-invasive approach for in vivo monitoring of drug distribution, but also a valuable approach for investigating the behavior of the formulation during development ex vivo. This is the suggestion of Stracke and colleagues, who report the use of MPM for studying the distribution of FONs within skin layers [197]. In particular, they prepared Texas red-loaded PLGA nanoparticles, starting from a fluorescein-conjugated polymer. Texas red, a sulforhodamine derivative and red fluorescent dye with low water solubility, λ_abs_ 596 nm, and λ_em_ 615 nm, was selected as model compound. A delivery study was performed by applying FONs (approx. average size 290 nm), embedded into a hydrogel, on excised human skin and observing the specimen over 5 h. PLGA nanoparticles showed green fluorescence as a response to fluorescein emission and remained stuck on the skin’s surface, unable to diffuse across the stratum corneum due to their large size. At the same time, the release of Texas red from the nanoparticles and its diffusion across epidermal layers was observed.

**Figure 7:**
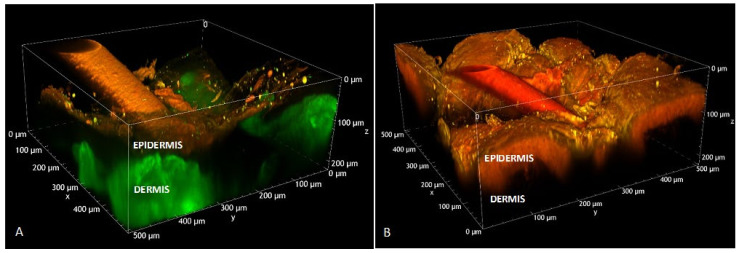
A 3D rendering of porcine skin samples, highlighting hair follicles. In panel (**A**), the reference sample is treated with saline solution, while in panel (**B**), we see the skin after contact with Nile red-loaded TPGS micelles. The retention of the Nile red within the epidermis is shown. (Reproduced from [200] under the Creative Commons Attribution License).

A similar study was performed using Nile red-loaded polymeric micelles (approx. average size 15 nm), prepared starting from d-α-tocopheryl polyethylene glycol 1000 succinate (TPGS) [200]. Nile red is a lipophilic fluorescent probe (λ_abs_ 552 nm; λ_em_ 635 nm), chosen as model for imiquimod, an immunostimulant drug approved for the treatment of actinic keratosis, a skin disease. The retention of imiquimod-loaded TPGS micelles was determined using isolated porcine skin. Then, in order to understand in-depth micelle behavior once in contact with tissue, Nile red micelles were placed in contact with isolated skin and analyzed by MPM. As depicted in Figure 7, the presence of Nile red is clearly observable across the whole epidermal thickness.

Furthermore, the shift of the fluorescence spectrum of the Nile red, collected within the tissue and indicating a change in the surrounded polarity, suggested that the fluorescent dyes had been released from the micelles. The same Nile red-loaded polymeric micelles were recently evaluated in ex vivo ocular tissue during a study [201].

### 7.4. Limits and Challenges

As we reviewed in this paper, FONs have many advantages and applications in drug delivery and bioimaging. However, it is important to note that several challenges prevail, which include the following: (i) The colloidal stability of FONs in biological media can pose a limitation and requires optimization, to avoid major leaching of the encapsulated dyes from the particle and to obtain a stable brightness and signal-to-noise ratio. (ii) Non-specific interactions in biological media can reduce the specificity to biomolecular targets in in vivo applications, requiring a fine-tuning of the surface chemistry to achieve the desired specificity. (iii) The scaled-up production of multifunctional FONs with low particle-to-particle and batch-to-batch variations are still challenging, and major efforts are being undertaken to optimize the fabrication techniques. (iv) A long-term in vivo toxicity evaluation is required for a successful application in the clinics, and over the years a more and more comprehensive data set will be available to guide future designs.

With the continuous progress being achieved in fluorophore design, the development of functional organic nanoparticle matrices, and the understanding of the mechanisms and interactions involved in the desired photophysical and physicochemical performance, we believe that fluorescent organic nanoparticles will find their clinical translation in different biomedical applications.

## 8. Future Trends, Directions, and Perspectives of FONs in Clinical Applications

As was presented in the review, FONs began to be used in the biological and clinical fields for various applications (e.g., imaging, sensing, diagnostics, drug delivery, and more). As many types of FONs are being developed, it seems that we are viewing just the tip of the iceberg when it comes to the possibilities of integrating FONs in the clinic. In what follows, we will present our perspectives on the future trends and directions of FONs in clinical applications: 1. Drug carriers—the ability to encapsulate various drugs (e.g., small molecules, antibodies, RNA, exosomes, etc.) will enable the use of FONs for targeted therapeutics (the FONs will target a specific tissue and release the drug in a localized area), and it will enable to cross challenging barriers (e.g., the BBB) and to provide new therapeutics to the brain. 2. XR (extended reality)—more and more hospitals adapt to XR operations, wherein they use special glasses to image the surgery. FONs will be used to bind to specific tissues, and by using such special glasses, the surgeon will be able to see the tissue and perform the precise removal of the tissue. 3. Diagnostic and sensing biocompatible FONs could be deployed to circulate in the body, and once they “detect” a specific molecule (related to a disease or health risk), they will start to emit light, so that they can be detected in the urine (or other methods). This may provide an “alarm” for specific pathological conditions. 

Further progress in the development of artificial intelligence (AI) and machine-based learning will also support the design of FONs with unique capabilities that are not available today [235].

## 9. Conclusions

FONs have been demonstrated to have major applications in biomedicine, as they provide fluorescent probes of high brightness for bioimaging. Meanwhile, they can also be exploited as targeted drug carriers and nanosensors. Overall, FONs appear to be versatile and useful tools in a clinical setting, still with great untapped potential, especially in the domain of theranostics, wherein imaging and diagnostic features are combined with therapeutic action. The complexity of such products, however, requires a careful selection of the carriers, their production methods, and their characterization. In this review, we provided an overview of the main types of FONs proposed for biomedical applications, their major requirements and desired properties, and illustrated the various production methods, including a practical guide to provide the reader with a useful tool for choosing the most suitable production method for a specific FON. In addition, we presented an overview of the various characterization methods and computational tools that provide invaluable support for the development and exploitation of new products based on FONs. Despite a wealth of reports, FONs will likely attract further increased scientific attention thanks to their versatile applicability and ability to operate as multifunctional nanotools, offering a tunable blend of chemical, physical, biological, and therapeutic properties.

## Figures and Tables

**Table 1:** Summary of the spectroscopic techniques which are commonly adopted for the investigation of the electronic properties of FONs. A = absorbance.

Spectroscopic Technique	Good Practices for Non-Scattering Samples	Good Practices for Scattering Samples	Information That Can Be Derived from the Spectroscopic Characterization
UV–vis-NIR Absorption	A < 2	Integrating sphere accessory; A < 2	Spectral range of absorbed light; aggregation effects [138]
Steady-state fluorescence	A < 0.1 to minimize inner filter effects	Double monochromators and optical filters; integrating sphere for QY; A < 0.1	Spectral range of emitted light; aggregation effects; fluorescence quantum yield [139]
Time resolved fluorescence	-	Front-face geometry/short-path cuvette	Lifetimes [139]
Fluorescence anisotropy	-	Scattering is detrimental: measurement of fluorescence anisotropy is compromised	Hydrodynamic radius [139]

**Table 2:** Fluorescence imaging techniques.

Technique	Excitation	Working Principle	Main Features	Ref.
Widefield Fluorescence Microscopy	UV–vis Linear excitation process	The entire sample is exposed to excitation light, and fluorescence is detected through a filter that only allows emitted light to reach the detector.	Very fast measurements Limited spatial resolution due to the collection of background fluorescence from unfocused region of the sample Limited to surface analysis	[157]
Confocal Microscopy	UV–vis Linear excitation process	Use of point illumination (focused beam) and of a pinhole to avoid out-of-focus detection of fluorescence to improve resolution.	Improved resolution with respect to widefield Limited penetration depth	[158]
Multiphoton Microscopy (MPM)	Red-NIR Non-linear excitation process Ultrashort laser pulses (fs laser)	Multiphoton excitation for deep 3D imaging (up to 1–2 mm).	High penetration depth (up to 1–2 mm) Intrinsic sub-micrometer 3D resolution Reduced photobleaching Reduced tissue damage	[159,160]
Fluorescence Lifetime Imaging Microscopy (FLIM)	UV–vis-NIR Linear/non-linear excitation process Pulsed excitation	The contrast is based on the difference between decay rates of different fluorophores.	High sensitivity Reduced background noise	[161]
Total Internal Reflection Fluorescence (TIRF)	UV–vis laser Linear excitation process	The evanescent wave at the interface between sample and an object (such as glass coverslip) is exploited.	High resolution (even beyond the diffraction limit) Penetration depth of hundreds of nm (mainly limited to the surface)	[162]
Near-Field Scanning Optical Microscopy (NSOM, SNOM)	UV–vis laser Linear excitation process Optical fiber probe having an aperture smaller than the wavelength of light	The sample is scanned at a small distance below the aperture, in the near field.	Optical resolution is limited only by the diameter of the aperture (even few nm)	[163]
Stimulated Emission Depletion (STED)	UV–vis-NIR Linear/non-linear excitation process Pulsed excitation. Two laser beams: typical Gaussian beam for excitation, doughnut-shaped de-excitation beam	The excitation beam creates excited-state population in a diffraction-limited region; the doughnut-shaped de-excitation beam deactivates all the fluorophores in the region except in the central area of the focal spot.	Resolution beyond the diffraction limit	[162,163]
Photoactivated Localization Microscopy (PALM) and Stochastic Optical Reconstruction Microscopy (STORM)	UV–vis Laser Linear excitation process	Both in PALM and STORM, fluorescence is collected from sparse subset of fluorophores in the sample. Many images, emanating from different sparse subsets, are collected and summed; the final image is reconstructed based on the localization of fluorophores. Typically, blinking fluorophores are used, such as some fluorescent proteins.	Resolution beyond the diffraction limit	[164,165]

**Table 3:** Summary of the current approaches for simulating the organic NPs and FONs.

Method	Time and Space Range	Principle	Typical Applications and Processes	Extensions	Ref.: Organic NPs and FONs
Model Hamiltonians and Essential State Models (ESMs)	Small systems with up to 100 electronic states and 103 atoms	Model electronic Hamiltonian with effective parameters constructed on the base of polar dyes or their fragments (e.g., [171]).	Strongly interacting systems (e.g., aggregates) with non-linear electronic and optical responses.	Linear and non-linear optical spectra with vibronic coupling and environment	Aggregate of dyes [147]
Quantum Mechanical (QM) and Density Functional Theory (DFT)	Small systems—with up to 103 atoms in the unit cell and tens of ps of simulation	Electronic-structure and/or ab initio methods, wherein the electronic degrees of freedom are accounted for and described through the electron wavefunction or density (DFT).	Systems wherein electronic, magnetic, and optical properties are relevant.	Ab initio molecular dynamics (aiMD): DFT is combined with the equations of motions to describe the evolution of nuclei Time-dependent (TD) DFT: employ linear response theory to compute, e.g., optical properties	Dyes for FONs with (TD)-DFT [172,173,174]
Atomistic Technique: All-Atoms (AAs) or United-Atoms (UAs)	Medium-to-large systems: 104 atoms and up to µs of simulations	Force fields (FFs) do not explicitly describe electrons and the systems are described by a set of “ball and springs”. FFs have been parameterized (and are refined) for many biologically relevant molecules, including lipids, nucleic acids, and proteins (e.g., AMBER, CHARMM OPLS-AA, etc.). FF potential is expressed in terms of bonding and non-bonding terms (e.g., electrostatics and Van der Waals).	Large systems, membranes, and small vesicles wherein the most important information is the molecular structure and statistical and dynamical properties.	Classical molecular dynamics: force field is used to compute forces that serve to describe atomic movements via the equations of motions	FONs self-assembly [172]: effect of cholesterol on liposomes [175,176], quatsomes (as membrane bilayer) [70,73,177], and surfactant micelles [178]
Coarse Grain (CG) Model	Large systems: 104–105 particles (~106 atoms) and µs of simulations	A set of atoms is grouped and treated as an interaction particle (or bead). This translates into a decrease in details, with the fading of the atomic resolution, but it allows for larger system sizes and longer time scales. MARTINI is among the most popular coarse-graining schemes [179].	Very large systems, whole membranes, and vesicles wherein the most important information is the overall structural arrangement and its dynamical properties.	Classical molecular dynamics: force field is used to compute forces that serve to describe CG particle movements via the equations of motions	Liposomes with hypericin drug [180] Micellization studies [181]. Self-assembly of cationic surfactant [182]

**Table 4:** Examples of organic nanoparticles and their application areas and current state of development.

**Type of Nanocarrier**	**Nanoparticle Components**	**Fluorophore**	**Application**	**Current State of Development**	**Ref.**
Liposomes	Stealth^®^ liposome (Doxil^®^)	Doxorubicin	Targeted therapeutic	Marketed, for human use	[195]
	Hydrogenated soybean phosphatidylcholine, cholesterol, 1,2-distearoyl-sn-glycero-3-phosphoethanolamine-N-methoxy–polyethylene glycol 2000	Cyanine 5 Rhodamine	Targeted therapeutic	Pre-clinical In vivo (mouse)	[196]
Quatsomes	Cholesterol Myristalkonium chloride	DiR	Bioimaging	Pre-clinical In vivo (mouse)	[71]
Polymeric nanoparticles	PLGA nanoparticles	Texas red	Drug delivery	Pre-clinical Ex vivo	[197]
	PLGA nanoparticles	Coumarin-6	Targeted therapeutic	Pre-clinical In vivo (mouse)	[198]
	PLGA nano- and micro-particles	Cresyl violet	Theranostic	Pre-clinical In vivo (mouse)	[199]
	Poly(ethyl methacrylate)-based polymer azide, bulky hydrophobic counterion, capture DNA-sequence (22–23 mer)	Rhodamine B, Atto665	Diagnostics	In vitro	[83]
Polymeric micelles	Diblock copolymer of PEG and a poly(methyl methacrylate) derivative	Indocyanine Green	Fluorescence-guided surgery	Clinical trials (Phase I/II)	[89]
	TPGS	Nile red	Drug delivery	Pre-clinical Ex vivo (pig skin)	[200]
	TPGS	Nile red	Drug delivery	Pre-clinical Ex vivo (pig ocular tissues)	[201]
Polymersomes	PEG_45_-PCL_60_-PNIPAM (Poly(N-isopropylacrylamide))_33_	BODIPY	Targeted therapeutic	In vitro Pre-clinical In vivo (mouse)	[94]
Polyelectrolytes	Hyaluronan–Doxorubicin complexes as drug carrier	Doxorubicin	Drug delivery	In vitro	[202]
Small- molecule FONs	Only the fluorophore	Variety of fluorophores	Imaging and particle tracking	In vitro	[203]

## Data Availability

Not applicable.

## Abbreviations

2PA	Two-Photon Absorption
AAs	All-Atoms
AiMDs	Ab initio Molecular Dynamics
ACQ	Aggregation-Caused Quenching
AIE	Aggregation-Induced Emission
CMC	Critical Micellar Concentration
CF	Compressed Fluid
CG	Coarse Grain or Coarse-Graining
DELOS	Depressurization of an Expanded Liquid Organic Solution
DFT	Density Functional Theory
DDS	Drug Delivery System
DPDs	Dissipative Particle Dynamics
FON	Fluorescent Organic Nanoparticle
FRET	Förster Resonance Energy Transfer
HME	Hot Melting Extrusion
HPH	High Pressure Homogenization
ICG	Indocyanine Green
MMs	Molecular Mechanics
MPM	MultiPhoton Microscopy
MPS	Microphysiological System
NLC	Nanostructured Lipid Carrier
NP	Nanoparticle
OoC	Organs-On-a-Chip
PDT	Photodynamic Therapy
PE	Polyelectrolyte
QY	Quantum Yield
SLN	Solid Lipid Nanoparticle
TD	Time Dependent
TPGS	D-α-tocopheryl Polyethylene Glycol 1000 Succinate
UAs	United-Atoms
